# Multimodal Electrophysiological Signals for Machine Learning-Aided Parkinson’s Disease Diagnosis

**DOI:** 10.3390/bios16070381

**Published:** 2026-07-13

**Authors:** Bo Jiang, Han Liu, Yuchen Ran, Yan Zhou, Keke Chen, Xiao Yang, Jiayuan Zhao, Mengxuan Hu, Boyan Fang, Guangying Pei

**Affiliations:** 1 School of Medical Science and Engineering, Beijing Institute of Technology, Beijing 100081, China; jiangbo2688@163.com (B.J.);; 2 Parkinson Medical Center, Beijing Rehabilitation Hospital, Capital Medical University, Beijing 100144, China; 3 Beijing Key Laboratory of Brain-Inspired Neural Engineering, School of Medical Science and Engineering, Beijing Institute of Technology, Beijing 100081, China

**Keywords:** Parkinson’s disease, multimodal signals, classification model, complementary information

## Abstract

Parkinson’s disease (PD) is a neurodegenerative disorder affecting motor and autonomic nervous system functions. In this study, six synchronized modalities—electroencephalography (EEG), electrocardiography (ECG), electromyography (EMG), respiration (Resp), photoplethysmography (PPG), and gait (Gait)—were recorded from 25 PD patients and 25 healthy controls. A Random Forest classifier was used to perform both unimodal and multimodal signal classification. Among unimodal models, ECG achieved the highest accuracy (84%), whereas the performance of multimodal combinations did not increase linearly with the number of modalities; integrating three or more complementary signals was sufficient to substantially improve classification. The full six-modality model achieved an accuracy of 95.00%, precision of 94.17%, recall of 97.14%, F1 score of 95.21%, and an AUC of 0.98. Incremental analysis further indicated that selecting key complementary modalities can maintain high classification performance while reducing equipment requirements, simplifying experimental procedures, and improving participant comfort, providing guidance for the development of efficient, non-invasive PD diagnostic tools.

## 1. Introduction

Parkinson’s disease (PD) is a progressive neurodegenerative disorder with a complex aetiology, clinically defined by motor symptoms such as resting tremor, rigidity and bradykinesia [1,2]. Furthermore, PD manifests as a multidimensional physiological dysfunction, affecting the central, autonomic and motor systems. Consequently, reliance on unimodal signals provides an incomplete picture of the disease pathology, limiting the objectivity and accuracy of clinical diagnosis [3,4]. Unraveling the multimodal physiological mechanisms of PD is essential not only for developing effective interventions but also for establishing the objective diagnostics required to advance personalized medicine and rationalize healthcare resource allocation [5,6].

The complex pathophysiology of PD is reflected in a range of physiological signals. Electroencephalography (EEG) studies have identified alterations in PD patients, including cortical slowing, indicated by changes in α-band relative power and the θ/α ratio, as well as increased β-band oscillations [7,8]. At the motor level, the characteristic resting tremor can be assessed using electromyography (EMG) [9]. Autonomic dysfunction is frequently observed through reduced heart rate variability (HRV), a metric under investigation as a potential early electrocardiogram (ECG) biomarker [10]. Furthermore, respiratory (Resp) complications are a documented contributor to mortality in PD [11], and severe gait (Gait) impairments, such as freezing of Gait, present in a significant subset of patients and are associated with reduced quality of life [12,13]. These physiological signatures provide a basis for computational diagnostic models. Previous research has employed machine learning algorithms, including Support Vector Machines (SVM) and Random Forests (RF), to classify PD patients from healthy controls (HC) using single-modality signals (e.g., EEG, ECG, Gait) or limited bimodal combinations [10,13,14]. A primary limitation of these approaches is that a single modality reflects only a specific dimension of the disease’s underlying pathology. For instance, EEG measures central nervous system activity, whereas Gait analysis assesses peripheral motor function. This constrained perspective is associated with an upper limit on classification accuracy, often reported to be below 90% [15,16,17]. Multimodal fusion, which integrates data from multiple sources, addresses this limitation by providing a more comprehensive representation of the patient’s condition, potentially leading to improved classification performance and robustness [18]. Recent AI-based studies have also explored physiological signals for PD-related assessment and related biomedical monitoring tasks. For example, CNN-based deep learning models have been applied to EEG-based PD diagnosis [19], while ECG/HRV-related studies have investigated autonomic dysfunction and cardiovascular regulation in PD [10]. In addition, multimodal biosignal classification and uncertainty-aware physiological monitoring have highlighted the importance of robust and reliable physiological signal modelling [20]. However, many existing studies have focused on single-modality signals, limited modality combinations, or task-specific monitoring scenarios. Systematic evaluation of the complementary value, relative contribution, and redundancy of multiple synchronized physiological and Gait modalities in PD classification remains limited. The relative diagnostic contribution and redundancy among different modalities have not been clearly defined. Furthermore, the effect on diagnostic performance resulting from the absence of a key modality has not been quantified. A systematic evaluation of these factors is necessary to inform the development of more efficient and clinically viable diagnostic systems [21].

To address this gap, this study utilizes a novel multimodal dataset constructed through the synchronous acquisition of five physiological signals, namely EEG, ECG, EMG, Resp, and photoplethysmography (PPG), alongside Gait data from patients with PD and HC. The analytical framework comprises three stages. First, inter-group statistical analysis is employed to identify significant physiological and Gait biomarkers, thereby elucidating the underlying pathophysiological mechanisms of PD. Second, an evaluation of three classical machine learning classifiers is conducted to determine the optimal model for multimodal disease identification. Finally, an incremental analysis is performed to investigate how classification performance evolves with the addition of each signal modality and to identify optimal combinations of physiological and Gait signals.

To support objective and non-invasive PD diagnosis from a multidimensional physiological perspective, this study was designed to construct a subject-level synchronized multimodal analysis framework integrating EEG, ECG, EMG, Resp, PPG, and Gait signals acquired from the same participants under a unified protocol. These modalities were used to characterize central cortical activity, autonomic and cardiorespiratory regulation, neuromuscular activation, peripheral pulse dynamics, and lower-limb motor execution. On this basis, inter-group statistical analysis was first performed to identify PD-related physiological and Gait features, followed by machine-learning classification using unimodal, full six-modality, and incremental multimodal fusion models. The incremental design and modality contribution analysis were further used to examine how different physiological systems complement each other in classification and to identify reduced modality combinations that preserve diagnostic performance while limiting signal redundancy and acquisition burden. Through this framework, this study aimed to clarify the diagnostic value of coordinated central and peripheral physiological information and provide evidence for efficient non-invasive auxiliary diagnosis of PD.

## 2. Materials and Methods

### 2.1. Participants

The recruitment of participants for this study was conducted by the Beijing Rehabilitation Hospital affiliated with Capital Medical University. Physicians screened potential participants using standardized clinical scales, excluding individuals with a history of neurological or psychiatric disorders, brain injuries, cardiovascular or respiratory diseases, as well as those with motor or cognitive impairments. A total of 25 patients with primary PD who met the diagnostic criteria set by the International Parkinson and Movement Disorder Society were included in this study. All patients with PD continued their usual antiparkinsonian medication regimen during the experiment and were assessed in the medicated “ON” state. No medication withdrawal or washout procedure was performed before signal acquisition. Additionally, 25 age- and sex-matched HC were recruited from the community (Table 1). All participants provided informed consent in accordance with the guidelines of the Declaration of Helsinki. Approval was obtained from the Medical Ethics Review Panel for Clinical Research Projects of Beijing Rehabilitation Hospital, Capital Medical University (approval number: Beikang Scientific Research Ethics Approval No. 2022bkky-140; date of approval: 15 March 2022).

### 2.2. Multi-Source Signal Acquisition

The synchronous acquisition of multimodal signals was accomplished through an integrated system. This system combined a proprietary laboratory apparatus for capturing EEG, ECG, EMG, Resp and PPG signals with a commercial device for Gait signal acquisition (Noraxon MyoMotion, Noraxon U.S.A. Inc., Scottsdale, AZ, USA) [22]. The experimental protocol was divided into two stages. Initially, participants remained seated for a six-minute period in a controlled laboratory setting to facilitate the collection of resting-state multimodal physiological data. Following this, participants wore Gait sensors on their thighs and calves and were instructed to complete three trials of walking along a straight 10 m path. The Gait data obtained from these three trials were averaged to obtain a representative dataset for subsequent analysis (specific parameters are provided in Table 2).

### 2.3. Multimodal Data Preprocessing

The detailed acquisition and post-acquisition preprocessing parameters for each modality are summarized in Table 2. Using MATLAB software (version R2023a; The MathWorks, Inc., Natick, MA, USA) and the EEGLAB toolbox (version 2022), EEG, ECG, EMG, PPG and Resp data were downsampled to 250 Hz to reduce data weight and improve computational efficiency, followed by a 50 Hz notch filter to remove power line interference. Subsequently, the EEG data were band-pass filtered between 0.1 and 45 Hz, and independent component analysis was employed to eliminate artifacts such as physiological noise from electrooculography and EMG. The ECG data were bandpass filtered between 0.5 and 40 Hz, and the R-peaks were detected using the findpeaks function. The generated CSV files were imported into Kubios HRV Scientific (version 4.1.2.1; Kubios Oy, Kuopio, Finland). The EMG signals were bandpass filtered between 0.5 and 45 Hz. The PPG signals were bandpass filtered between 0.5 and 35 Hz. Finally, the Resp signals were bandpass filtered between 0.1 and 3 Hz. The Gait signal comprises both positive and negative components corresponding to each anatomical joint angle movement. The neutral anatomical position for each joint is defined as zero degrees, with left rotation and left flexion represented as negative values, while right rotation and right flexion are represented as positive values. To ensure consistency in data representation, all negative values should be uniformly converted to positive values.

### 2.4. Data Feature Extraction

For each signal, feature extraction is carried out following these steps: (1) The power spectral density of the EEG is computed using the Welch method. The preprocessed continuous EEG recording of each participant was divided into 2000 ms windows with 50% overlap solely for spectral estimation. A periodogram was calculated for each window, and the resulting spectral estimates were averaged across all windows to obtain the final participant-level power spectral density features. The frequency bands analyzed were delta (0.5–4 Hz), theta (4–8 Hz), alpha (8–13 Hz), beta (13–30 Hz), and gamma (30–45 Hz). Although EEG was recorded from 32 channels, the channel-wise power spectral density estimates were aggregated to obtain one whole-brain power spectral density (PSD) value for each frequency band at the participant level. Accordingly, the initial EEG feature set comprised only five participant-level features—δ, θ, α, β, and γ—rather than 160 channel-by-frequency features. The overlapping windows were used only as intermediate units in the Welch procedure and were not treated as independent samples for machine-learning classification. (2) The EMG signals focus on the electrophysiological characteristics of muscle contractions. Five EMG features were extracted, including root mean square amplitude (RMS), mean frequency, frequency centroid, kurtosis, and peak value. (3) ECG signals were used to calculate average heart rate variability-related features, including mean heart rate, maximum heart rate, minimum heart rate, standard deviation of RR intervals (SDNN), very low-frequency power (VLF, 0–0.04 Hz), low-frequency power (LF, 0.04–0.15 Hz), and high-frequency power (HF, 0.15–0.40 Hz). (4) The periodic contraction (systole) and relaxation (diastole) of the heart are prerequisites for the generation of the PPG. Regarding PPG characteristics, the main parameters calculated were pulse rate, mean pulse-to-pulse interval, standard deviation of pulse-to-pulse intervals, and pulse rate variability. (5) For the Resp signal, respiratory rate was extracted to characterize respiratory rhythm regulation. (6) For the Gait signal, lower-limb joint-angle features were extracted, including left knee flexion, right knee flexion, left ankle dorsiflexion, and right ankle dorsiflexion.

### 2.5. Diagnostic Model Construction

Based on the between-group statistical analysis, only features showing significant differences between the PD and HC groups were included in the subsequent classification analysis. For EEG, these comprised the whole-brain α- and γ-band PSD features, which were combined with statistically significant features derived from the other modalities. Before model construction, all statistically significant features included in the classification analysis were normalized to reduce the influence of scale differences among heterogeneous modalities. After normalization, each participant corresponded to one subject-level feature vector, and all subsequent data splitting and model validation procedures were performed at the subject level.

In this study, multimodal fusion was performed at the feature level. Specifically, the normalized subject-level features extracted from different modalities were concatenated to construct unimodal, multimodal, and incremental modality-combination feature sets. Feature-level fusion was selected because the sample size was relatively small and the extracted physiological features were interpretable and heterogeneous across modalities. Compared with more complex deep fusion strategies, this approach reduces model complexity and overfitting risk while allowing the contribution of each modality and feature to be analyzed.

RF-based feature importance was calculated using the built-in impurity-based importance measure, which reflects the mean decrease in Gini impurity contributed by each feature across all decision trees. To estimate modality-level contribution, the feature importance values of all selected features belonging to the same modality were summed and normalized across modalities. This grouped feature-importance analysis was used as an exploratory interpretation of which modalities contributed most to the multimodal RF classification model.

Based on features extracted from multi-source physiological and Gait signals, this study employed three machine learning algorithms, including SVM, eXtreme Gradient Boosting (XGBoost), and RF, to construct PD classification models [23,24,25]. After feature extraction, each participant corresponded to one subject-level feature vector, and all subsequent data splitting procedures were performed at the subject level. A stratified random sampling strategy was used to divide all participants into a training set comprising 80% of the participants and an independent test set comprising the remaining 20%, while maintaining an approximately balanced ratio of patients with PD to HC in both datasets. All physiological and Gait features from the same participant were always assigned to the same data subset, and there was no subject overlap between the training and test sets.

Model hyperparameters were optimized on the training set using grid search, and the independent test set was not involved in this process. To evaluate the stability of model performance, ten-fold cross-validation was conducted within the training set. Specifically, the training set was stratified into 10 non-overlapping subsets. In each iteration, one subset was used as the validation set, while the remaining nine subsets were used for model training. This process was repeated 10 times, and the mean and standard deviation of each evaluation metric were calculated. Ten-fold cross-validation was used as the primary validation strategy because it provides a balance between training sample size and performance stability. Because the primary objective was to compare the diagnostic performance of single-modal models with the full six-modality model, 95% confidence intervals were reported for this primary performance analysis. The algorithm comparison and incremental modality-combination analyses were considered exploratory and were reported as mean ± SD to summarize performance trends. As an additional subject-level validation strategy, leave-one-out cross-validation (LOOCV) was performed for the primary comparison between the single-modal models and the full six-modality model. During LOOCV, model development procedures were conducted using only the training subjects in each iteration, while the held-out subject was used only for validation. The LOOCV results were used as supplementary internal validation to assess whether the main performance trend was consistent under a different subject-level validation strategy. The independent test set was used only for the final evaluation of model generalization performance. This strategy ensured that the test set was not involved in model construction at any stage, thereby avoiding data leakage and reducing the risk of overfitting.

Feature selection in this study was based on the between-group statistical analysis described in the Statistical Analysis section, and only statistically significant features were retained for classification. No additional algorithm-based feature selection method was applied. No additional feature-scaling procedure was performed on the original full feature set; instead, normalization was applied only to the selected statistically significant features before model construction. Importantly, feature selection and normalization were conducted within the training data rather than on the whole dataset in advance. During cross-validation, the selected feature subset and normalization parameters were determined using only the training subjects in each fold and then applied to the corresponding held-out validation subjects. For the independent test evaluation, the feature subset and normalization parameters were determined using only the training set and then applied to the independent test set, thereby avoiding data leakage.

Model performance was evaluated using accuracy, precision, recall, F1 score, receiver operating characteristic (ROC) curves, and the area under the curve (AUC). Accuracy represents the proportion of correctly classified participants among all participants. Precision indicates the proportion of participants who were truly PD among those predicted as PD. Recall, also known as sensitivity, reflects the model’s ability to correctly identify patients with PD. The F1 score is the harmonic mean of precision and recall. ROC curves and AUC were used to comprehensively evaluate the discriminative ability of the models in distinguishing patients with PD from HC. In the incremental multimodal feature experiment, RF was used as the classifier, and different types of subject-level features were progressively integrated according to predefined modality combinations. All modality combinations followed the same data splitting and 10-fold cross-validation procedures described above. To comprehensively compare the contribution of different modality combinations, the accuracy of each combination was calculated on the reserved independent test set, and the modality combinations were ranked according to their cross-validated test performance. The performance ranking of each modality combination was based on predictions from unseen samples, thereby objectively reflecting differences in the generalization ability of different feature combinations.

Hyperparameter tuning was performed using a nested grid-search strategy within the 10-fold cross-validation framework. In each outer cross-validation fold, the held-out test fold was not involved in feature selection, hyperparameter optimization, model training, or model selection. Because multiple classifiers and different modality combinations were evaluated in this study, the optimal hyperparameters were selected independently for each classifier, each modality combination, and each training fold; therefore, a single fixed hyperparameter configuration was not used across all models.

The predefined hyperparameter search spaces were as follows. For the SVM model, the penalty parameter (C) was searched from 0.1 to 5.0 with a step size of 0.1, and the kernel coefficient (gamma) was searched from 10^−4^ to 10^−1^ on a logarithmic scale. For the RF model, the number of decision trees (n_estimators) was searched from 10 to 100 with a step size of 10; the maximum tree depth (max_depth) and the maximum number of features considered at each split (max_features) were both searched from 1 to 10; and the minimum number of samples required to split an internal node (min_samples_split) was searched from 2 to 10. For the XGBoost model, the learning rate (eta) was searched from 10^−2^ to 10^0^ on a logarithmic scale; the number of weak learners (n_estimators) was searched from 10 to 400; the maximum tree depth (max_depth) was searched from 1 to 10; the minimum child weight (min_child_weight) was searched from 0 to 10; and the minimum loss reduction required for further node splitting (gamma) was searched from 0 to 2. The final model performance was reported as the average result across all independent outer test folds.

### 2.6. Statistical Analysis

All statistical analyses were performed using SPSS version 26.0 software (IBM Corp, Armonk, NY, USA) and MATLAB software (The MathWorks, Inc., Natick, MA, USA). First, the normality of each variable was assessed using the Shapiro–Wilk test. For between-group comparisons of demographic characteristics, gender was analyzed using the chi-square (χ^2^) test, whereas age and other non-normally distributed continuous variables were compared using the Mann–Whitney U test. For scale data, as well as multimodal physiological signals and Gait features, normally distributed variables were analyzed using independent-samples *t*-tests, while non-normally distributed variables were analyzed using the Mann–Whitney U test. All statistical tests were two-sided, and *p* < 0.05 was considered statistically significant.

## 3. Results

### 3.1. Characteristics of Multimodal Physiological Signals for PD

**Figure 1:**
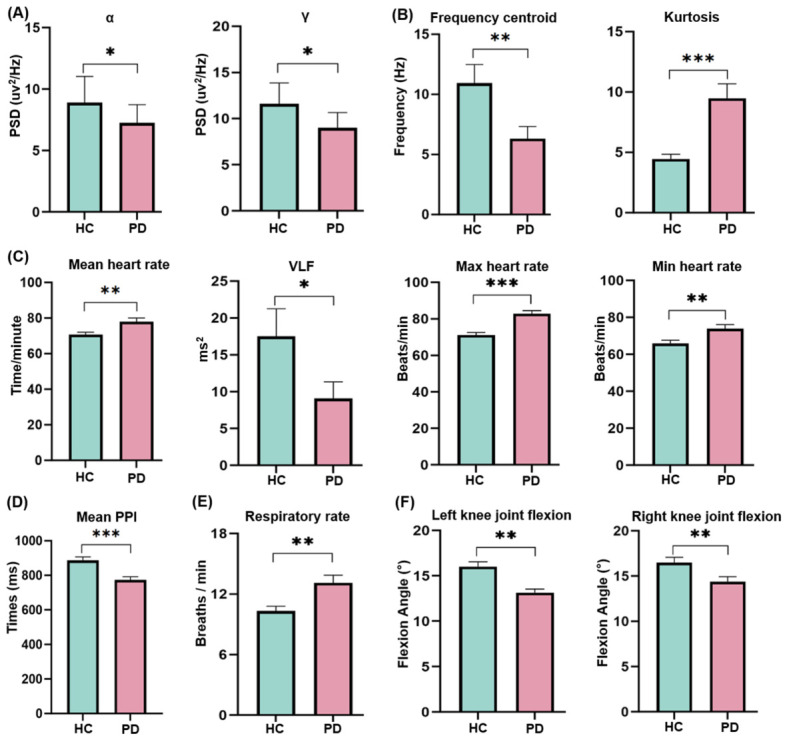
Multimodal Physiological and Gait Characteristics of PD. (**A**) EEG power spectral density (PSD) in alpha and gamma bands for PD patients and HC. (**B**) EMG features: Frequency centroid and kurtosis for PD patients and HC. (**C**) Heart rate-related features: Mean heart rate, very low frequency (VLF), maximum heart rate, and minimum heart rate for PD patients and HC. (**D**) PPG feature: Mean pulse-to-pulse interval (PPI) for PD patients and HC. (**E**) Resp feature: Resp rate comparison between PD patients and HC. (**F**) Gait features: Left and right knee joint flexion for PD patients and HC (* *p* < 0.05, ** *p* < 0.01, *** *p* < 0.001).

The analysis of the results indicated that patients with PD exhibited significant differences in six physiological signals, as illustrated in Figure 1.

In the EEG signals, the α-band power spectral density (*U* = 205.500, *p* = 0.037) and γ-band power spectral density (*U* = 187.000, *p* = 0.016) in PD patients were significantly lower than those observed in the HC group (Figure 1A). However, no significant differences were detected in the β-, θ-, and δ-band power spectral densities (all *p* > 0.05). In the EMG signals, the frequency centroid of PD patients (*U* = 178.500, *p* = 0.009) was significantly lower compared to that of the HC group, while the kurtosis (*U* = 104.500, *p* < 0.001) was significantly higher than that in the HC group (Figure 1B). Nevertheless, the root mean square amplitude, mean frequency, and EMG peak did not exhibit significant differences between PD patients and HC (*p* > 0.05). In the ECG signals, the average heart rate (*t* = −2.926, *p* = 0.002), maximum heart rate (*U* = 91.000, *p* < 0.001), and minimum heart rate (*t* = −2.914, *p* = 0.008) of patients with PD were significantly higher than those of the HC group. Conversely, the very low frequency (*U* = 200.000, *p* = 0.029) was significantly lower in PD patients compared to HC (Figure 1C). However, the standard deviation of RR intervals, as well as low-frequency power and high-frequency power, did not exhibit significant differences between PD patients and HC (*p* > 0.05). In the PPG signals, the mean pulse-to-pulse interval of PD patients (*U* = 110.000, *p* < 0.001) was significantly shorter than that of the HC group (Figure 1D). However, pulse rate, the standard deviation of pulse-to-pulse intervals and pulse rate variability, did not exhibit significant differences between PD patients and HC groups (*p* > 0.05). In the Resp signals, the Resp rate (*U* = 175.000, *p* = 0.008) of PD patients was significantly higher than that of the HC group, as illustrated in Figure 1E. In the analysis of Gait signals, it was found that left knee flexion (*U* = 176.500, *p* = 0.009) and right knee flexion (*U* = 166.000, *p* = 0.004) in patients with PD were significantly lower than those in the HC group (Figure 1F). However, no significant differences were observed in left ankle joint dorsiflexion and right ankle joint dorsiflexion between PD patients and HC (*p* > 0.05).

### 3.2. Diagnostic Performance of Single-Modal and Multimodal Models

**Figure 2:**
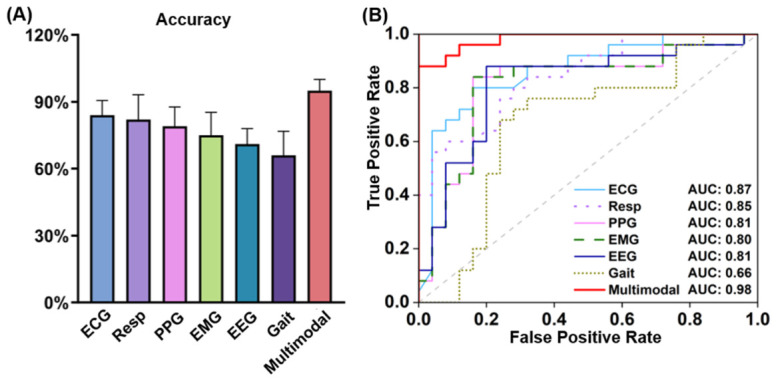
Comparison of accuracy and ROC curves for single-modal and multimodal disease recognition models. (**A**) Comparison of accuracy for the single-modal and six-modality multimodal models (error bars: standard deviation, *n* = 50). (**B**) ROC curve comparison for the single-modal and six-modality multimodal.

An initial evaluation of three machine learning classifiers—SVM, XGBoost, and RF—on multimodal features identified RF as the top performer, achieving the highest recognition accuracy (see Figure S1 and Table S1 in the Supplementary Materials for details). Accordingly, RF was selected to construct and compare a series of unimodal and multimodal disease recognition models. Figure 2A shows the classification accuracies of unimodal and multimodal disease recognition models. The integrated multimodal model achieved the highest accuracy (95.00%), markedly outperforming all unimodal models. Among the unimodal classifiers, ECG exhibited the highest accuracy (84.00%), followed by Resp (82.00%) and PPG (79.00%), whereas Gait achieved the lowest accuracy (66.00%). Figure 2B presents the ROC curves and corresponding AUC values for unimodal and multimodal models. The curves illustrate each model’s ability to discriminate PD patients from HC, showing the trade-off between the true positive rate (sensitivity) and the false positive rate (1 − specificity) across different thresholds. The multimodal model achieved the highest AUC (0.98), followed by the unimodal models. Among the unimodal models, ECG and Resp achieved the highest AUCs (0.87 and 0.85, respectively), whereas the Gait model exhibited the lowest AUC (0.66).

Table 3 summarizes the ten-fold cross-validation performance of the single-modal and full six-modality models. The six-modality model achieved the highest accuracy (95.00%), precision (94.17%), recall (97.14%), and F1 score (95.21%), outperforming all single-modal models. Among the single-modal models, ECG, Resp, and PPG showed relatively higher performance, whereas EEG and Gait showed lower performance. An additional subject-level LOOCV analysis was performed for the single-modal and six-modality models. As shown in Table 4, the LOOCV results showed a generally consistent performance trend with the ten-fold cross-validation results, with the six-modality model showing the highest overall performance.

### 3.3. Modality Contribution and Incremental Evaluation of Multimodal Combination Diagnostic Models

To further examine which modalities contributed most to the multimodal classification results, RF-based feature importance was analyzed at the modality level. The contribution of each modality was calculated by summing the normalized importance values of all selected features belonging to that modality. ECG showed the highest contribution to the classification model, followed by Resp, Gait, EEG, EMG, and PPG. The modality-level contribution values were 0.23 for ECG, 0.20 for Resp, 0.19 for Gait, 0.15 for EEG, 0.13 for EMG, and 0.10 for PPG. These results indicate that cardiovascular autonomic regulation and respiratory rhythm features were the dominant contributors, while Gait, EEG, EMG, and PPG provided additional complementary information for PD classification.

**Figure 3:**
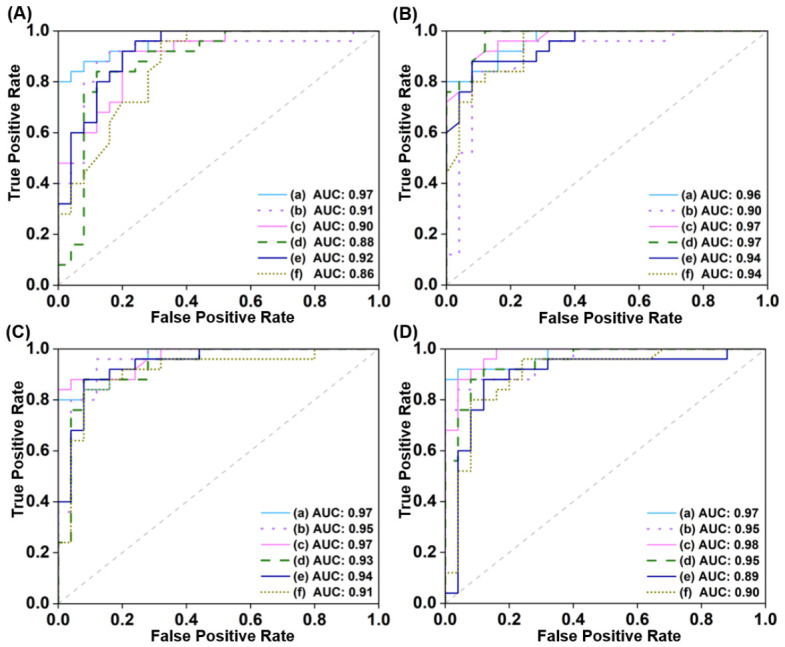
ROC curves of the top six incremental multimodal models ranked by classification accuracy. (**A**) ROC curves of two-modality combinations. (**B**) ROC curves of three-modality combinations. (**C**) ROC curves of four-modality combinations. (**D**) ROC curves of five-modality combinations. (The detailed information of each modality combination (a–f) is provided in Supplementary Materials for Figure 3).

In addition, the incremental modality-combination analysis was used as an ablation-like analysis to evaluate how different modality combinations affected classification performance. The following presents the results of the incremental analysis of multimodal fusion. ROC analysis was performed for the top six models ranked by classification accuracy under two-, three-, four-, and five-modality combinations. As shown in Figure 3, most incremental multimodal models showed AUC values generally above 0.90.

Incremental analysis of multimodal combinations was performed for two- to five-modality models (Figure 3A–D, Tables S2–S5 in the Supplementary Materials). Among the two-modality combinations, ECG + Resp achieved the highest accuracy (89.00%) and AUC (0.97), while ECG + EMG and EEG + ECG reached accuracies of 86.00%, with AUCs of 0.91 and 0.90, respectively (Figure 3A, Table S2). For three-modality combinations, ECG + Resp + Gait achieved the highest accuracy (94.00%) and an AUC of 0.96, whereas EEG + EMG + PPG and ECG + Resp + PPG both attained accuracies of 90.00%, each with an AUC of 0.97 (Figure 3B, Table S3). Among the four-modality combinations, EEG + ECG + EMG + Resp achieved the highest accuracy (93.00%) and an AUC of 0.97, with EEG + ECG + Resp + Gait and ECG + Resp + PPG + Gait also showing high accuracies of 91.00% (Figure 3C, Table S4). For the five-modality combinations, EEG + ECG + EMG + Resp + Gait attained the highest accuracy (93.00%) and AUC (0.97) (Figure 3D, Table S5); however, its accuracy did not exceed that of the top three-modality model.

Table 5 presents the performance of various EEG-based classification models, including those that integrate EEG with other modalities. The models were evaluated using accuracy, precision, recall, and F1 score, with standard deviations reported. Accuracy increased progressively from 71.00% in EEG1 to 95.00% in EEG6, with the multi-modality model (EEG6: EEG + ECG + EMG + Resp + PPG + Gait) achieving the highest accuracy. Precision ranged from 77.44% (EEG1) to 94.17% (EEG6), showing improvement as additional modalities were incorporated. Recall similarly increased with the number of modalities, from 79.55% (EEG1) to 97.14% (EEG6). F1 scores followed the same trend, ranging from 71.89% in EEG1 to 95.21% in EEG6, with multi-modality models consistently outperforming single- or limited-modality models across all metrics.

Table 6 presents the performance of various Gait-based classification models, evaluated using accuracy, precision, recall, and F1 score, with standard deviations reported. Accuracy increased from 66.00% in Gait1 to 95.00% in Gait6, with the multi-modal model (Gait6: EEG + ECG + EMG + Resp + PPG + Gait) achieving the highest accuracy. Precision ranged from 73.83% (Gait1) to 94.17% (Gait6), showing improvement as additional modalities were incorporated. Recall similarly increased from 68.07% (Gait1) to 97.14% (Gait6), indicating better identification of true positives with more integrated modalities. F1 scores followed the same trend, from 63.74% in Gait1 to 95.21% in Gait6, with the multi-modal model consistently achieving the highest performance across all metrics.

## 4. Discussion

In this study, after integrating five synchronous physiological signals and one Gait signal, including EEG, ECG, EMG, Resp, PPG, and Gait, the classification accuracy of the RF model reached 95.00%, with an AUC of 0.98, indicating that multimodal feature fusion can effectively enhance PD recognition performance. Importantly, incremental analysis showed that the improvement in model performance is not simply proportional to the number of modalities included. For instance, the accuracy of the dual-modality combination of ECG and Resp reached 89.00%, while the accuracy of the three-modality combination further increased to 94.00%, approaching the performance of the complete six-modality model. However, adding additional modalities beyond three did not always yield substantial gains, suggesting a potential diminishing returns effect in multimodal fusion [26,27]. These findings indicate that the diagnostic value of multimodal models depends not only on the number of included signals but also on the degree of complementary information among the modalities.

The discriminative features identified in this study were associated with the multisystem involvement of PD. Patients with PD showed lower EEG α- and γ-band PSD than HC, which may indicate altered cortical electrophysiological regulation and is consistent with previous EEG findings in PD [14,28]. The increased heart rate, decreased VLF power, and shorter PPG-derived pulse-to-pulse interval may reflect abnormal autonomic cardiovascular regulation and peripheral pulse dynamics, in line with previous evidence of cardiac and autonomic dysfunction in PD [10,29]. The increased respiratory rate may be related to altered respiratory rhythm regulation, as respiratory-system alterations have been reported in PD [11]. In addition, the lower EMG frequency centroid, higher EMG kurtosis, and reduced knee flexion during Gait may reflect abnormal neuromuscular activation and impaired lower-limb motor execution, corresponding to typical motor manifestations and Gait abnormalities in PD [13,30]. Overall, these features jointly covered central cortical activity, autonomic and respiratory regulation, peripheral pulse dynamics, neuromuscular activation, and motor execution, thereby supporting the complementary value of multimodal signal integration.

The RF-based modality contribution analysis further supported the complementary value of multimodal fusion. ECG contributed the most to the multimodal RF model, followed by Resp, Gait, EEG, EMG, and PPG, with modality-level contribution values of 0.23, 0.20, 0.19, 0.15, 0.13, and 0.10, respectively. This finding is consistent with previous evidence that cardiovascular autonomic dysfunction and respiratory abnormalities are common non-motor manifestations of PD [11,31]. Therefore, the relatively high contributions of ECG and Resp may reflect the sensitivity of cardiovascular autonomic and respiratory rhythm features to PD-related multisystem alterations. Notably, although Gait showed the lowest unimodal classification accuracy, its modality-level contribution reached 0.19 in the multimodal model, suggesting that Gait features provided complementary motor-execution information when combined with autonomic and respiratory features. In contrast, PPG showed the lowest contribution, which may be related to partial redundancy with ECG-derived cardiovascular information.

From a clinical perspective, the discriminative features identified in this study should be interpreted within the broader context of multisystem involvement in PD. Although PD is primarily a neurodegenerative disorder involving central dopaminergic and related neural circuits, autonomic and cardiorespiratory dysfunctions are important non-motor manifestations of the disease. Previous studies have emphasized that cardiac dysfunction and cardiovascular autonomic abnormalities are closely related to PD and may even appear before typical motor symptoms, suggesting the potential value of cardiovascular monitoring in PD assessment [32]. In this context, the ECG-derived features identified in the present study, including increased mean, maximum, and minimum heart rate and reduced VLF power, may reflect altered cardiovascular autonomic regulation. Respiratory dysfunction has also been reported in PD and may involve respiratory rhythm regulation, respiratory muscle impairment, upper airway dysfunction, and central control mechanisms [31]. Therefore, the increased respiratory rate observed in patients with PD may be related to altered respiratory regulation and impaired cardiopulmonary control. The shorter PPG-derived pulse-to-pulse interval may further reflect peripheral pulse dynamics and vascular-related cardiovascular regulation, with possible partial overlap with ECG-derived cardiac information.

EEG, EMG, and Gait features provide complementary information from central neural activity to motor execution. Previous quantitative EEG studies have reported altered cortical oscillatory activity in PD, including changes in spectral power and slowing of cortical rhythms [33]. Thus, the reduced α- and γ-band power observed in this study may reflect PD-related alterations in cortical electrophysiological regulation. In contrast, EMG and Gait features are more closely related to neuromuscular activation and motor-output abnormalities. These features may correspond to characteristic motor manifestations of PD, such as bradykinesia, rigidity, tremor, reduced movement amplitude, and Gait impairment [12]. Accordingly, the EMG frequency centroid and kurtosis may indicate altered neuromuscular activation patterns, whereas reduced knee flexion during Gait may reflect impaired lower-limb motor execution. Taken together, these findings suggest that the discriminative features are not isolated statistical markers, but correspond to clinically relevant central neural, autonomic/cardiorespiratory, peripheral vascular, neuromuscular, and motor-output abnormalities in PD.

Nevertheless, the modality contribution results should be interpreted as model- and dataset-specific discriminative contributions rather than a biological hierarchy of PD mechanisms. Although the full six-modality model achieved the highest classification performance, collecting all modalities may increase device complexity, preparation time, synchronization requirements, and participant burden. Therefore, the incremental analysis should be regarded as an exploratory assessment of modality complementarity and clinical feasibility, rather than evidence that any reduced set of modalities can replace the full multimodal framework. The proposed multimodal framework should be considered a potential non-invasive decision-support approach for PD assessment, rather than a replacement for clinical diagnosis.

These modality-level contribution differences were generally consistent with the unimodal classification results. In unimodal models, ECG and Resp exhibit the highest classification accuracies, reaching 84.00% and 82.00%, respectively, outperforming EMG, EEG, PPG, and Gait. Notably, the AUC of ECG in unimodal models reaches 0.87, suggesting that heart rate-related time-frequency features, such as reductions in VLF components, may possess strong discriminative capabilities in distinguishing PD patients from HC [31,32]. This finding aligns with previous evidence indicating that PD patients often experience autonomic nervous dysfunction, and abnormalities in physiological indicators such as heart rate variability may emerge early in the disease, even preceding typical motor symptoms [32]. In contrast, the unimodal performance of Gait signals is the lowest, with an accuracy of 66.00%. This may be attributed to the relatively simple Gait task employed in this study, which involved short-distance straight walking, as well as the limited set of Gait features primarily based on single joint angle measurements. Under short-distance, obstacle-free straight walking conditions, characteristic Gait instability of PD patients, particularly in mild to moderate cases, may not have been sufficiently elicited [30].

Further analysis of EEG- and Gait-related models provides supplementary evidence for the complementary roles of central nervous system activity and motor execution in PD classification. In the EEG series, unimodal EEG performance is limited, with an accuracy of 71.00% and an F1 score of 71.95%, likely because EEG primarily reflects the electrophysiological activity of the central nervous system, but provides limited or indirect information regarding autonomic regulation and peripheral motor execution [34,35]. Although EEG can reflect PD-related alterations in cortical electrophysiological activity, it does not directly quantify nigrostriatal neurodegeneration, and its classification performance is highly dependent on the experimental paradigm and feature representation. Chaturvedi et al. used 256-channel resting-state EEG and extracted 79 quantitative EEG features, yet the classification accuracies of different models ranged from only 56.0% to 78.0% [36]. In addition, Saikia et al. reported an accuracy of 62% for the unimodal EEG model, which was lower than the 73% achieved by the unimodal EMG model [37]. Therefore, the 71.00% accuracy of the EEG model in the present study falls within the range reported in previous studies. Its lower performance than ECG and respiration may be related to more prominent autonomic and cardiorespiratory abnormalities in the present cohort, as well as the relatively limited representation of EEG features. As additional modalities (ECG, EMG, Resp, Gait, and PPG) are integrated, the performance of EEG-related multimodal models progressively improves, reaching 95.00% accuracy and 95.21% F1 score in the six-modality model. Similarly, in the Gait series, unimodal Gait exhibits the lowest performance (accuracy 66.00%, F1 score 63.74%), Gait mainly reflects macroscopic kinematics, making it difficult to capture individual physiological differences and easily influenced by factors such as tasks, clothing, feature selection, and disease duration, resulting in limited discriminative power in this study. However, integrating ECG, Resp, EEG, and EMG markedly enhances classification [38,39]. These findings indicate that, while EEG and Gait alone have limited discriminative power, they provide important complementary information in multimodal fusion: EEG captures central nervous activity, whereas Gait reflects peripheral motor execution outcomes [40]. Combining these with autonomic and muscular signals allows the model to detect PD-related abnormalities across multiple pathophysiological dimensions. The results of this study further support previous findings that diagnostic models integrating multiple physiological signals generally outperform those based on a single signal, thereby providing a more comprehensive and accurate assessment for PD [41,42].

To further contextualize the proposed framework, we compared our results with representative studies on machine learning-based PD assessment using physiological or multimodal signals. Previous studies have commonly focused on single physiological signals or limited multimodal combinations. For example, Guo et al. integrated EEG, ECG, PPG, and respiratory signals and achieved an accuracy of 96.03% using a multimodal SVM model [43], supporting the diagnostic potential of combining heterogeneous physiological signals. Compared with their study, the present work further incorporated EMG and Gait signals and systematically evaluated unimodal, full six-modality, and incremental multimodal fusion models. In addition, Zhang et al. developed a multimodal dataset consisting of accelerometer signals, EEG, EMG, and skin conductance signals for freezing-of-Gait detection in PD [12]. Different from their focus on freezing-of-Gait detection, the present study aimed at PD-versus-HC classification by integrating central cortical activity, autonomic and cardiorespiratory regulation, neuromuscular activation, peripheral pulse dynamics, and lower-limb motor execution features. The full six-modality model achieved an accuracy of 95.00% and an AUC of 0.98, while the reduced ECG + Resp + Gait combination reached an accuracy of 94.00%. These findings suggest that synchronized multimodal integration can improve PD classification and that selected complementary modalities may preserve high classification performance while reducing signal redundancy and acquisition burden.

This study has several limitations that should be addressed in future research. First, due to the relatively small sample size, this study is exploratory in nature and the findings should be regarded as preliminary, which may limit the generalizability and robustness of the model. Future studies should validate the proposed multimodal framework in larger samples, independent cohorts, and multicenter datasets. Second, all patients with PD were assessed in the medicated “ON” state. This design was adopted to ensure patient comfort and safety and to reduce severe tremor-related artifacts, large motor fluctuations, and unstable Gait performance during multimodal signal acquisition. However, antiparkinsonian medications, especially dopaminergic medications, may influence EEG, EMG, autonomic regulation, and Gait characteristics; therefore, their effects on the present findings cannot be excluded. Future studies should compare patients in both “ON” and “OFF” medication states to further clarify medication-related effects. Third, the Gait assessment paradigm in this study is relatively simple; future research could introduce more challenging walking tasks, such as dual-task walking, turning, or variable-speed walking, to better capture PD-related Gait impairments [44]. Fourth, although the RF model demonstrated strong classification performance, the current fusion strategy primarily relies on simple integration at the feature level, which may not fully capture complex interactions among modalities. Future research could adopt advanced fusion approaches, such as cross-modal attention mechanisms, modality weighting strategies, or feature selection algorithms, to identify the most informative and complementary modality combinations while reducing redundant information [45,46]. These strategies may enhance model interpretability, reduce computational burden, and facilitate the clinical translation of multimodal PD diagnostic models. In addition, recent advances in soft and bioactive flexible bioelectronics may support the development of more comfortable wearable multimodal acquisition systems for long-term physiological monitoring in future PD studies [47].

## 5. Conclusions

Multimodal models outperformed unimodal signals in PD classification, mainly because they integrated complementary information from central neural activity, autonomic regulation, and peripheral motor execution. The full six-modality RF model achieved the highest classification accuracy of 95.00%, outperforming the best unimodal ECG model, which achieved an accuracy of 84.00%. EEG provides central electrophysiological information, ECG and Resp reflect autonomic and cardiopulmonary regulation, whereas EMG and Gait capture neuromuscular activity and motor execution outcomes. Incremental analysis indicated that simply adding more modalities did not always improve performance, suggesting that the physiological complementarity among modalities is more important than the number of signals included. Notably, the three-modality combination of ECG + Resp + Gait achieved an accuracy of 94.00%, approaching the performance of the full six-modality model. These findings support the use of integrated central and peripheral indicators for developing efficient, non-invasive diagnostic tools for PD.

## Figures and Tables

**Table 1:** The demographics and clinical scales of subjects.

	PD	HC	*p*-Value
Sex (M/F)	25 (13/12)	25 (13/12)	1.00 ^a^
Age (SD)	62.2 (6.6)	64.7 (4.3)	0.41 ^b^
H&Y (SD)	2.12 (0.46)	-	-
UPDRS III (SD)	21.88 (7.72)	-	-
MMSE (SD)	27.88 (1.39)	28.08 (1.49)	0.42 ^b^
MoCA (SD)	24.68 (2.92)	25.24 (2.87)	0.45 ^c^

M/F: Male/Female; SD: Standard Deviation; H&Y: Hoehn & Yahr stage; UPDRS III: Movement Disorders Society-Unified Parkinson’s Disease Rating Scale-Part III (severity of motor symptoms); MMSE: Mini-Mental State Examination; MoCA: Beijing version of the Montreal Cognitive Assessment. ^a^ Chi-square test, ^b^ Mann–Whitney U test, ^c^ Independent sample *t*-test.

**Table 2:** Acquisition and preprocessing parameters of multimodal physiological signals.

Signal Type	Sampling Frequency (Hz)	Acquisition Setup (Positions/Parameters)	Acquisition Duration	Post-Acquisition Processing
EEG	1000	Whole-brain 32-channel acquisition Reference: Bilateral mastoids	6 min	Downsampled to 250 Hz; 50 Hz notch filtering; 0.1–45 Hz band-pass filtering
ECG	1000	The III lead bonding method is adopted	6 min	Downsampled to 250 Hz; 50 Hz notch filtering; 0.5–40 Hz band-pass filtering
EMG	1000	Biceps brachii. The electrodes are placed in a three-point manner	6 min	Downsampled to 250 Hz; 50 Hz notch filtering; 0.5–45 Hz band-pass filtering
PPG	1000	Finger-clip PPG sensor	6 min	Downsampled to 250 Hz; 50 Hz notch filtering; 0.5–35 Hz band-pass filtering
Resp	1000	Chest and abdominal respiratory belts	6 min	Downsampled to 250 Hz; 50 Hz notch filtering; 0.1–3 Hz band-pass filtering
Gait	100	Sensors were placed on the thigh, shank, and foot	6 min (Three trials of a 10 m walking test)	The average value of three walking trials was calculated

**Table 3:** Classification performance of single-modal and multi-modal models using ten-fold cross-validation.

Model	Accuracy (%)	Precision (%)	Recall (%)	F1 Score (%)
ECG	84.00 ± 6.64 [79.48–88.52]	82.31 ± 10.58 [73.21–91.41]	82.31 ± 8.51 [68.35–96.27]	81.57 ± 10.07 [72.10–91.04]
Resp	82.00 ± 11.18 [71.44–92.56]	90.17 ± 10.07 [84.18–96.14]	82.29 ± 10.80 [74.35–90.24]	83.48 ± 10.28 [75.00–91.95]
PPG	79.00 ± 8.72 [71.89–86.11]	75.62 ± 12.19 [67.66–83.58]	85.64 ± 10.78 [77.52–93.77]	79.49 ± 12.59 [70.00–88.98]
EMG	75.00 ± 10.25 [67.27–82.73]	82.17 ± 10.73 [67.17–97.16]	73.19 ± 11.56 [61.16–85.22]	74.48 ± 9.66 [67.20–81.77]
EEG	71.00 ± 7.00 [65.57–76.43]	77.44 ± 12.19 [70.82–84.06]	79.55 ± 9.70 [69.57–89.53]	71.89 ± 7.71 [64.69–79.17]
Gait	66.00 ± 10.77 [53.75–78.25]	73.83 ± 11.72 [61.10–86.66]	68.07 ± 11.63 [65.61–70.53]	63.74 ± 11.06 [52.67–74.81]
Multi -modal	95.00 ± 5.00 [90.17–99.83]	94.17 ± 5.17 [88.63–99.71]	97.14 ± 5.72 [92.83–99.88]	95.21 ± 5.09 [90.92–99.50]

The above data are presented as mean ± SD [95% CI]. SD: standard deviation; CI: confidence interval. The 95% confidence intervals were calculated from fold-level cross-validation performance using a t-distribution-based approach. Multi-modal: EEG + ECG + EMG + Resp + PPG + Gait.

**Table 4:** Subject-level leave-one-out validation performance of single-modal and six-modality models.

Model	Accuracy (%)	Precision (%)	Recall (%)	F1 Score (%)
ECG	81.58 ± 5.47 [70.95–90.00]	85.06 ± 7.51 [68.18–98.10]	77.59 ± 8.50 [61.64–94.22]	80.80 ± 6.11 [68.49–90.34]
Resp	81.42 ± 6.34 [68.85–92.00]	84.47 ± 9.65 [64.63–98.31]	78.39 ± 8.76 [61.96–92.46]	80.87 ± 7.10 [66.01–92.16]
PPG	78.30 ± 6.97 [64.95–91.05]	76.28 ± 9.08 [54.00–91.49]	80.70 ± 11.67 [57.40–98.75]	77.86 ± 8.37 [59.27–92.91]
EMG	69.96 ± 8.04 [54.00–81.05]	77.92 ± 13.70 [51.25–99.98]	66.26 ± 8.63 [50.35–83.17]	65.17 ± 9.29 [50.00–83.00]
EEG	77.12 ± 7.59 [58.95–89.05]	73.75 ± 9.65 [54.17–91.02]	83.13 ± 9.73 [59.09–96.61]	77.89 ± 8.67 [57.92–90.91]
Gait	73.84 ± 6.72 [62.00–87.05]	72.48 ± 8.12 [58.89–90.70]	67.18 ± 10.56 [51.03–89.83]	72.26 ± 8.09 [57.17–87.32]
Multi -modal	94.36 ± 3.07 [88.00–99.82]	93.96 ± 4.35 [85.71–99.56]	94.50 ± 4.25 [84.92–99.95]	94.14 ± 3.26 [86.65–99.68]

Data are presented as mean ± SD [95% CI]. SD: standard deviation; CI: confidence interval; LOOCV: leave-one-out cross-validation. The 95% confidence intervals were calculated using percentile-based bootstrap resampling. Multi-modal: EEG + ECG + EMG + Resp + PPG + Gait.

**Table 5:** Performance evaluation of classification models based on EEG modalities.

Model	Accuracy (%)	Precision (%)	Recall (%)	F1 Score (%)
(EEG1)	71.00 (±7.00)	77.44 (±12.19)	79.55 (±9.70)	71.89 (±7.71)
(EEG2)	86.00 (±12.00)	86.67 (±11.18)	80.62 (±12.85)	82.81 (±12.43)
(EEG3)	90.00 (±10.99)	87.50 (±12.18)	90.17 (±10.27)	88.71 (±11.16)
(EEG4)	93.00 (±6.53)	91.67 (±10.54)	94.64 (±8.64)	92.71 (±7.63)
(EEG5)	93.00 (±6.41)	93.50 (±10.01)	93.14 (±8.59)	92.81 (±6.73)
(EEG6)	95.00 (±5.00)	94.17 (±5.17)	97.14 (±5.72)	95.21 (±5.09)

The above data type is mean (±SD). SD: Standard Deviation; (EEG1): EEG; (EEG2): EEG + ECG; (EEG3): EEG + EMG + PPG; (EEG4): EEG + ECG + EMG + Resp; (EEG5): EEG + ECG + EMG + Resp + Gait; (EEG6): EEG + ECG + EMG + Resp + PPG + Gait.

**Table 6:** Performance evaluation of classification models based on Gait modalities.

Model	Accuracy (%)	Precision (%)	Recall (%)	F1 Score (%)
(Gait1)	66.00 (±10.77)	73.83 (±11.72)	68.07 (±11.63)	63.74 (±11.06)
(Gait2)	85.00 (±12.85)	90.17 (±11.17)	81.71 (±11.31)	84.41 (±13.06)
(Gait3)	94.00 (±6.64)	93.00 (±11.40)	93.81 (±10.66)	93.11 (±9.80)
(Gait4)	91.00 (±8.31)	92.14 (±10.86)	89.31 (±11.84)	90.36 (±10.00)
(Gait5)	93.00 (±6.41)	93.50 (±10.01)	93.14 (±8.59)	92.81 (±6.73)
(Gait6)	95.00 (±5.00)	94.17 (±5.17)	97.14 (±5.72)	95.21 (±5.09)

The above data type is mean (±SD). SD: Standard Deviation; (Gait1): Gait; (Gait2): Gait + ECG; (Gait3): Gait + ECG + Resp; (Gait4): Gait + ECG + Resp + EEG; (Gait5): Gait + ECG + Resp + EEG + EMG; (Gait6): EEG + ECG + EMG + Resp + PPG + Gait.

## Data Availability

The data that support the findings of this study are available on request from the corresponding author, but restrictions apply to the availability of these data, which were used under license for the current study, and so are not publicly available.

## Supplementary Materials

The following supporting information can be downloaded at: https://www.mdpi.com/article/10.3390/bios16070381/s1, Figure S1: Comparison of the classification performance of different machine learning algorithms using multimodal features. (A) Classification accuracy of RF, SVM, and XGBoost. (B) ROC curves of the three machine learning algorithms; Table S1: Classification performance of different machine learning algorithms using multimodal features; Table S2: Classification performance of the top six two-modality combinations for PD; Table S3: Classification performance of the top six three-modality combinations for PD; Table S4: Classification performance of the top six four-modality combinations for PD; Table S5: Classification performance of the top six five-modality combinations for PD.
