# Supplementary material for: Multimodal Electrophysiological Signals for Machine Learning-Aided Parkinson’s Disease Diagnosis
Source: Biosensors (Basel). 2026 Jul 13;16(7):381. doi: 10.3390/bios16070381 (PMC13407023; doi:10.3390/bios16070381)
Supplement: Supplementary file 1 [file biosensors-16-00381-s001.zip › biosensors-4374320-supplementary.pdf]

Supplementary Materials

Comparison of Machine Learning Classification Methods

Using multimodal signal features that differed significantly between the PD and HC groups, we compared three machine learning classifiers. RF achieved an accuracy of 95.00%, compared with 90.00% for SVM and 87.00% for XGBoost (Figure S1A). The corresponding AUC values were 0.98, 0.96, and 0.96, respectively (Figure S1B). RF showed the best overall classification performance and was therefore selected for subsequent unimodal, full six-modality, and incremental multimodal classification analyses. Detailed results are provided in Table S1.

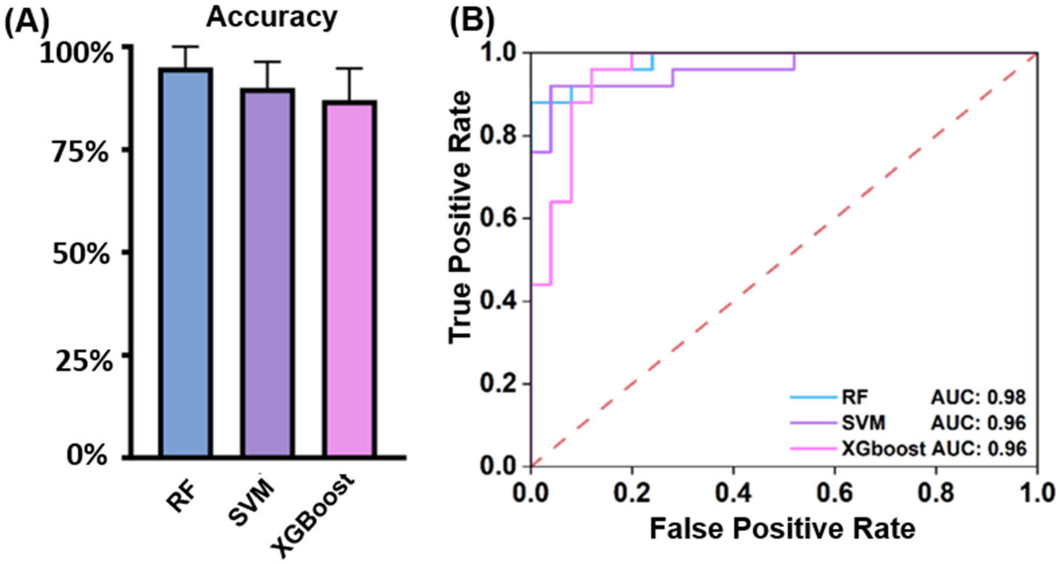

Figure S1. Comparison of the classification performance of different machine learning algorithms using multimodal features. (A) Classification accuracy of RF, SVM, and XGBoost. (B) ROC curves of the three machine learning algorithms.

Table S1. Classification performance of different machine learning algorithms using multimodal features.

Model	Accuracy (%)	Precision (%)	Recall (%)	F1 Score (%)
RF	95.00 (± 5.00)	94.17 (± 5.17)	97.14 (± 5.72)	95.21 (± 5.09)
SVM	90.00 (± 6.33)	90.83 (± 12.05)	87.31 (± 11.55)	88.59 (± 9.82)
XGBoost	87.00 (± 7.81)	88.33 (± 13.10)	87.71 (± 10.54)	87.04 (± 8.31)

The above data type is mean(±SD). SD: Standard Deviation.

Details of the Modality Combinations Shown in Figure 3

Figure 3 in the main manuscript presents the top six two-, three-, four-, and five-modality combinations ranked by classification accuracy. The modality combinations corresponding to labels (a)–(f) in each panel, together with their classification performance, are listed below.

Two-modality combinations shown in Figure 3A:

(a) ECG + Resp;

(b) ECG + EMG;

(c) EEG + ECG;

(d) ECG + Gait;

(e) Resp + PPG;

(f) ECG + PPG.

Table S2. Classification performance of the top six two-modality combinations for PD.

Model	Accuracy (%)	Precision (%)	Recall (%)	F1 Score (%)
(a)	89.00 (± 7.00)	89.57 (± 11.43)	87.81 (± 11.11)	88.23 (± 9.17)
(b)	86.00 (± 10.20)	87.17 (± 12.29)	83.31 (± 9.79)	84.73 (± 12.80)
(c)	86.00 (± 12.00)	86.67 (± 11.18)	80.62 (± 12.85)	82.81 (± 12.43)
(d)	85.00 (± 12.85)	90.17 (± 11.17)	81.71 (± 11.31)	84.41 (± 13.06)
(e)	85.00 (± 8.06)	87.74 (± 11.01)	85.71 (± 11.84)	85.12 (± 7.55)
(f)	84.00 (± 8.00)	83.08 (± 12.79)	85.64 (± 10.78)	83.74 (± 9.52)

The above data type is mean(±SD). SD: Standard Deviation.

Three-modality combinations shown in Figure 3B:

(a) ECG + Resp + Gait;

(b) EEG + EMG + PPG;

(c) ECG + Resp + PPG;

(d) ECG + EMG + Resp;

(e) EEG + ECG + Resp;

(f) EEG + EMG + Resp.

Table S3. Classification performance of the top six three-modality combinations for PD.

Model	Accuracy (%)	Precision (%)	Recall (%)	F1 Score (%)
(a)	94.00 (± 6.64)	93.00 (± 11.40)	93.81 (± 10.66)	93.11 (± 9.80)
(b)	90.00 (± 10.99)	87.50 (± 12.18)	90.17 (± 10.27)	88.71 (± 11.16)
(c)	90.00 (± 6.33)	89.33 (± 11.53)	89.81 (± 11.32)	89.09 (± 9.23)
(d)	90.00 (± 7.75)	87.98 (± 12.90)	91.31 (± 11.79)	89.14 (± 10.58)
(e)	89.00 (± 9.44)	89.33 (± 12.09)	87.88 (± 12.99)	87.97 (± 11.84)
(f)	88.00 (± 10.50)	89.33 (± 10.62)	88.90 (± 10.35)	88.64 (± 10.34)

The above data type is mean(±SD). SD: Standard Deviation.

Four-modality combinations shown in Figure 3C:

(a) EEG + ECG + EMG + Resp;

(b) EEG + ECG + Resp + Gait;

(c) ECG + Resp + PPG + Gait;

(d) EMG + Resp + PPG + Gait;

(e) EEG + ECG + Resp + PPG;

(f) EEG + Resp + PPG + Gait.

Table S4. Classification performance of the top six four-modality combinations for PD.

Model	Accuracy (%)	Precision (%)	Recall (%)	F1 Score (%)
(a)	93.00 (± 6.53)	91.67 (± 10.54)	94.64 (± 8.64)	92.71 (± 7.63)
(b)	91.00 (± 8.31)	92.14 (± 10.86)	89.31 (± 11.84)	90.36 (± 10.00)
(c)	91.00 (± 7.00)	90.83 (± 12.05)	89.31 (± 11.84)	89.70 (± 10.40)
(d)	89.00 (± 9.44)	96.57 (± 6.98)	83.71 (± 15.39)	88.79 (± 10.00)
(e)	88.00 (± 6.00)	86.55 (± 12.26)	89.31 (± 11.84)	87.26 (± 9.26)
(f)	88.00 (± 10.77)	88.38 (± 10.43)	91.14 (± 12.74)	88.41 (± 10.92)

The above data type is mean(±SD). SD: Standard Deviation.

Five-modality combinations shown in Figure 3D:

(a) EEG + ECG + EMG + Resp + Gait;

(b) EEG + ECG + EMG + Resp + PPG;

(c) ECG + EMG + Resp + PPG + Gait;

(d) EEG + ECG + Resp + PPG + Gait;

(e) EEG + ECG + EMG + PPG + Gait;

(f) EEG + EMG + Resp + PPG + Gait.

Table S5. Classification performance of the top six five-modality combinations for PD.

Model	Accuracy (%)	Precision (%)	Recall (%)	F1 Score (%)
(a)	93.00 (± 6.41)	93.50 (± 10.01)	93.14 (± 8.59)	92.81 (± 6.73)
(b)	91.00 (± 7.00)	89.33 (± 11.53)	91.81 (± 11.18)	90.20 (± 9.79)
(c)	89.00 (± 7.00)	91.67 (± 10.54)	87.21 (± 10.68)	88.21 (± 8.30)
(d)	88.00 (± 11.66)	85.31 (± 13.55)	91.31 (± 11.79)	87.48 (± 10.06)
(e)	87.00 (± 11.00)	86.31 (± 12.83)	87.31 (± 11.55)	86.17 (± 10.97)
(f)	86.00 (± 12.00)	90.00 (± 11.13)	81.31 (± 12.05)	84.91 (± 12.64)

The above data type is mean(±SD). SD: Standard Deviation.
